# A Comparative Analysis of SegFormer, FabE-Net and VGG-UNet Models for the Segmentation of Neural Structures on Histological Sections

**DOI:** 10.3390/diagnostics15182408

**Published:** 2025-09-22

**Authors:** Igor Makarov, Elena Koshevaya, Alina Pechenina, Galina Boyko, Anna Starshinova, Dmitry Kudlay, Taiana Makarova, Lubov Mitrofanova

**Affiliations:** 1 Almazov National Medical Research Centre, St. Petersburg 197341, Russia; doctormakarovia@gmail.com (I.M.); koshevaya_eg@almazovcentre.ru (E.K.); pechenina_aa@almazovcentre.ru (A.P.); boyko_ga@almazovcentre.ru (G.B.); makarova_ta@almazovcentre.ru (T.M.); mitrofanova_lb@almazovcentre.ru (L.M.); 2 Department of Mathematics and Computer Science, St-Petersburg State University, St. Petersburg 199034, Russia; 3 Department of Pharmacology, Institute of Pharmacy, I.M. Sechenov First Moscow State Medical University, Moscow 119991, Russia; d624254@gmail.com; 4 Institute of Immunology FMBA of Russia, Moscow 115522, Russia; 5 Faculty of Bioengineering and Bioinformatics, Lomonosov Moscow State University, Moscow 119991, Russia

**Keywords:** digital pathology, convolutional neural networks, segmentation of histological images, segmentation of nerve fibres, visual transformers

## Abstract

**Background:** Segmenting nerve fibres in histological images is a tricky job because of how much the tissue looks can change. Modern neural network architectures, including U-Net and transformers, demonstrate varying degrees of effectiveness in this area. The aim of this study is to conduct a comparative analysis of the SegFormer, VGG-UNet, and FabE-Net models in terms of segmentation quality and speed. **Methods:** The training sample consisted of more than 75,000 pairs of images of different tissues (original slice and corresponding mask), scaled from 1024 × 1024 to 224 × 224 pixels to optimise computations. Three neural network architectures were used: the classic VGG-UNet, FabE-Net with attention and global context perception blocks, and the SegFormer transformer model. For an objective assessment of the quality of the models, expert validation was carried out with the participation of four independent pathologists, who evaluated the quality of segmentation according to specified criteria. Quality metrics (precision, recall, F1-score, accuracy) were calculated as averages based on the assessments of all experts, which made it possible to take into account variability in interpretation and increase the reliability of the results. **Results:** SegFormer achieved stable stabilisation of the loss function faster than the other models—by the 20–30th epoch, compared to 45–60 epochs for VGG-UNet and FabE-Net. Despite taking longer to train per epoch, SegFormer produced the best segmentation quality, with the following metrics: precision 0.84, recall 0.99, F1-score 0.91 and accuracy 0.89. It also annotated a complete histological section in the fastest time. Visual analysis revealed that, compared to other models, which tended to produce incomplete or excessive segmentation, SegFormer more accurately and completely highlights nerve structures. **Conclusions:** Using attention mechanisms in SegFormer compensates for morphological variability in tissues, resulting in faster and higher-quality segmentation. Image scaling does not impair training quality while significantly accelerating computational processes. These results confirm the potential of SegFormer for practical use in digital pathology, while also highlighting the need for high-precision, immunohistochemistry-informed labelling to improve segmentation accuracy.

## 1. Introduction

Modern medicine is increasingly oriented towards standardisation, acceleration, and enhanced accuracy in diagnostics, with digital pathology playing a pivotal role in this transformation. This approach entails digitising histological specimens and subsequently processing them using computer algorithms, enabling pathologists to identify structures, perform morphometric analyses, and, in some cases, detect patterns that may not be apparent to the human eye.

Conventional image analysis techniques—such as thresholding, morphological operations, and texture analysis—have generally shown limited effectiveness when applied to histological data. The main challenges include high variability in staining, the presence of artefacts, and the intricate morphology of biological structures.

The first notable study to propose an alternative methodology was conducted by Sjöström et al. (1999) [1]. The authors employed a partially fully connected neural network (NN) with two hidden layers (28–32 and 4–5 neurons, respectively) to automate cell counting in histological sections. The input layer processed image fragments of 24 × 24 or 48 × 48 pixels. The results demonstrated that the NN achieved accuracy comparable to manual counting (R^2^ ≈ 0.85–0.94) while offering a six-fold increase in processing speed (~25 s versus ~3 min for an expert). This work marked a key milestone in the adoption of neural networks within digital pathology.

At the turn of the 2000s, fully connected architectures were progressively replaced by convolutional neural networks (CNNs). One of the earliest studies in this area was conducted by Malon et al. [2], who employed a CNN similar to LeNet-5 to analyse histological images of biopsy specimens from breast and gastric cancer patients, despite the limited amount of training data available. The model demonstrated high accuracy in recognising colonic crypts (area under the curve, AUC ≈ 1) and achieved efficiency comparable to that of pathologists in identifying mitotic figures (κ = 0.40 versus 0.45–0.64 for physicians), while also yielding good results in segmenting ring-shaped cells.

These and subsequent studies have significantly advanced the field: as of 2025, more than 150 publications have been devoted to the application of CNNs in histological image processing.

CNNs are a specialised class of neural networks (NNs) designed for processing structured data, such as images. Their defining feature is the use of convolutional layers, which apply sets of filters (convolution kernels) to the input data, scanning the image and detecting local patterns. At the initial stages, the network identifies simple features such as edges, textures, and geometric shapes (e.g., lines and circles). In deeper layers, the CNN integrates these elementary features into more complex representations, including cell nuclei, tissue-specific morphological characteristics, and even entire histological patterns.

The effectiveness of CNNs in feature extraction can be attributed to two principal mechanisms:

- Hierarchical learning: The network automatically constructs a hierarchy of features, beginning with low-level characteristics such as edges and textures, and progressing to high-level representations such as object shapes and their spatial relationships. This process is analogous to the functional principles of the human visual cortex.
- Transformation invariance: Through pooling operations, a CNN becomes robust to minor shifts, scaling, and distortions in images, while preserving the essential features [3].

CNN training is performed via backpropagation, in which the filter weights are iteratively adjusted to minimise the discrepancy between the network’s predictions and the ground-truth data. During the validation stage, the trained model applies the learned patterns to previously unseen images, thereby demonstrating its capacity for generalisation. Variations among CNN architectures (e.g., LeNet-5, ResNet, and U-Net) are determined by factors such as network depth, the types of layers used (convolutional, pooling, and fully connected), and the manner in which these layers are interconnected. This flexibility enables the architecture to be tailored to specific tasks, ranging from cell segmentation to tumour classification.

## 2. Materials and Methods

For the study, 64 histological sections stained with haematoxylin and eosin were selected. To enhance the model’s accuracy and its capacity to differentiate between various tissue types, the dataset included a diverse range of histological materials: 14 prostate tissue sections, 18 sections of the aorta and pulmonary artery, 5 sections of clitoral and vulvar tissue, 16 myocardial sections with epicardial tissue, 6 colon sections, and 5 liver tissue sections.

The study was conducted in accordance with the Declaration of Helsinki and was approved by the Ethics Committee of the Almazov National Medical Research Centre (Protocol No. 10-22 dated 3 October 2022). Written informed consent was obtained from all participants whose data could be potentially identifiable.

All samples were digitised using an Aperio AT2 histological scanner (Leica Biosystems, Vista, CA, USA) at 20× magnification, corresponding to approximately 300–400× magnification under an optical microscope. The resulting digital images were processed using Aperio ImageScope software v. 12.4 (Leica Biosystems Imaging, Vista, CA, USA), in which nerve fibres and ganglia were manually annotated. The annotation results were stored in XML files containing the precise coordinates of each identified object.

The next stage of data processing involved the implementation of two distinct approaches. For 60% of the samples, only regions containing nerve fibres and adjacent tissues were extracted, yielding fragments measuring 1024 × 1024 pixels. For the remaining 40%, the entire histological sections were tiled into non-overlapping squares of 1024 × 1024 pixels. For each selected area, a corresponding binary mask was generated, in which a value of 0 (black) denoted background tissue and a value of 1 (white) indicated the presence of nerve structures.

This processing resulted in a comprehensive training dataset comprising more than 75,000 image–mask pairs (original histological fragment and corresponding mask). For validation, 10% of the images were randomly selected to provide an unbiased estimate of model performance during training. In addition, model testing was performed on a specialised dataset containing seven myocardial scans, from which 5600 labelled images were generated to ensure independent evaluation on previously unseen histological material. To optimise computational efficiency, all images were resized from 1024 × 1024 pixels to 224 × 224 pixels. This conversion was performed for the following reasons:

- To significantly accelerate data processing.
- To reduce the computational load during the model training stage.
- To prepare the model for more effective application to real-world data.
- To ensure compliance with standard input dimensions used in most modern CNN architectures.

**Figure 1:**
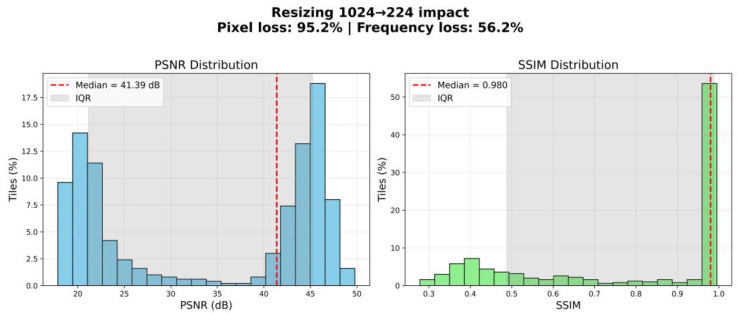
Estimating information loss during image resizing.

It should be noted that the scaling process preserved the key morphological characteristics of the tissues, as confirmed by visual quality control of the images following resizing. To verify that resizing did not compromise morphological integrity, we performed a quantitative evaluation of information loss. Specifically, we compared original and resized–upscaled tiles using peak signal-to-noise ratio (PSNR) and structural similarity index (SSIM). The analysis demonstrated a median PSNR of 41.39 dB (IQR: 21.14–45.24) and a median SSIM of 0.980 (IQR: 0.488–0.989). Although resizing theoretically implied a 95.2% reduction in pixel count and an estimated 56.3% loss of frequency content, the preserved structural similarity remained high (Figure 1).

These findings indicate that the scaling process retained the essential morphological features of the tissues. In particular, large anatomical structures such as nerve fibres remained clearly distinguishable, ensuring the suitability of the resized dataset for the intended segmentation task. Thus, resizing to 224 × 224 pixels represents a justified balance between computational feasibility and preservation of biologically relevant information.

**Figure 2:**
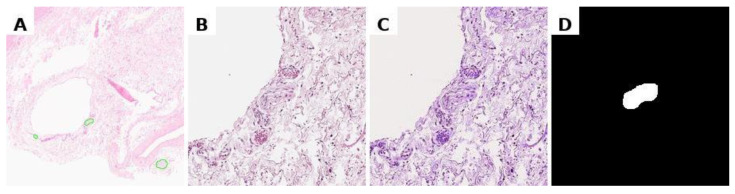
Preprocessing data for training. (**A**) marking of the svs file in Aperio ImageScope, (**B**) obtaining a histological image tile with a nerve at specified coordinates, resizing to 224 × 224 pixels; (**C**) normalisation of the histological image; (**D**) binary segmentation mask.

To minimise the impact of staining variability in histological preparations, image normalisation was performed using the Macenko method, which standardises tissue colour representation while preserving morphological features. The procedure involved: (1) converting the images to optical density space; (2) extracting the stain matrix using singular value decomposition (SVD); and (3) normalising stain concentrations relative to a reference sample (Figure 2). Binary masks were generated from polygonal annotations on whole-slide images using a custom Python script with OpenSlide. For each region of interest, the annotated area was cropped with a margin, and the corresponding binary mask was created by filling the annotation polygon (values 0/255). A full implementation of this method is briefly described in Supplementary Material (“Script for preprocessing svs-files to create mask–original image pairs.html”).

**Figure 3:**
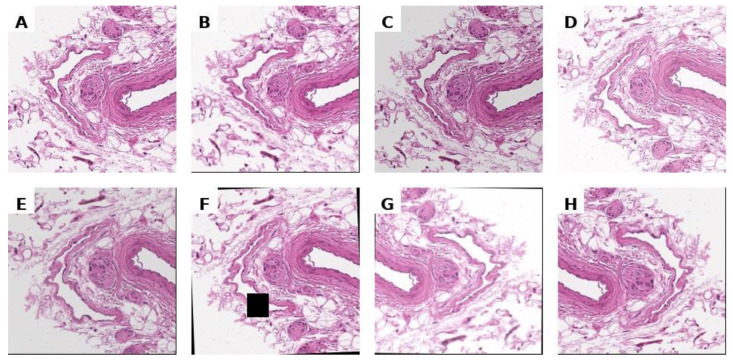
Unnormalized histological image (**A**) and its arbitrary augmentations (**B**–**H**).

To increase the diversity of the training dataset and mitigate overfitting, we applied real-time data augmentation using the Albumentations library. The augmentation pipeline comprised: (1) geometric transformations, including random horizontal and vertical reflections as well as small-angle rotations up to ±15°; (2) photometric modifications, specifically random brightness and contrast adjustments; and (3) morphological distortions via elastic deformations and coarse dropout (random patch removal). All transformations were applied stochastically during training, ensuring that each input image was potentially unique across epochs. This strategy improved model robustness to variability in tissue morphology, staining, and acquisition artefacts without requiring duplication of the original samples. Examples of images after the application of augmentation are shown in Figure 3.

Three neural network architectures were employed for the segmentation of nerve fibres in histological images: VGG-UNet, FabE-Net, and SegFormer. The models were trained on a graphics processing unit (GPU) with 15 GB of RAM, using interactive environments provided by Kaggle and Google Colab (Python 3.13). The computational framework was primarily based on PyTorch 2.8 and TensorFlow 2.20 for deep learning. Data preprocessing and augmentation utilised Albumentations 0.0.10, OpenCV 4.12, scikit-image 0.25, and PIL 11.3. Additional numerical and utility operations were implemented with NumPy 2.3, scikit-learn 1.7, tqdm 4.67, and the Python standard library (os). Whole-slide image handling was performed with OpenSlide 3.4. Visualization and plotting relied on Matplotlib 3.10 and Seaborn 0.13.

### 2.1. The Model VGG-UNet

The VGG-UNet model is based on a modified U-Net architecture incorporating a VGG-type encoder. It accepts a 224 × 224 × 3 colour image as input and outputs a 224 × 224 × 1 binary mask representing the predicted location of nerve fibres.

The encoder is implemented using a pre-trained VGG-16 convolutional network comprising five blocks, each containing 2–3 Conv2D layers with ReLU activation, followed by a MaxPooling2D layer for progressive spatial downsampling. The encoder’s weights are initialised with parameters pre-trained on ImageNet, which accelerates training and enhances segmentation performance when working with limited datasets.

The decoder mirrors the encoder’s structure and consists of the following components: transposed convolutions (Conv2DTranspose) for resolution restoration; feature concatenation (skip connections) with the corresponding encoder layers; two consecutive convolutional layers (Conv2D) with BatchNormalization and LeakyReLU activation; and Dropout layers for regularisation.

The final layer of the model is a 1 × 1 convolution with sigmoid activation, which projects the output onto a probability map for the nerve fibre/ganglion class (the model architecture diagram is provided in Supplementary Material, file Architecture of VGG-UNet.png).

The primary loss function was a composite metric designed to account not only for the degree of mask overlap but also for the impact of false positive predictions—an aspect of particular importance when analysing histological images containing fine structures. The overall loss function comprised the following components:

Binary Cross-Entropy (BCE): Measures the local pixel-wise difference between the predicted mask and the ground-truth mask (formulas for all loss functions are available in the Formulas.html in Supplementary Material).

IoU Loss (Jaccard loss): Evaluates the quality of the intersection between the predicted and true regions.

False Positive Penalty: Introduces an additional penalty for false positive pixels, i.e., background tissue incorrectly classified as nerve fibre. The penalty is scaled by a factor of α = 2.5.

The composite loss function used in training is expressed as follows:

(1) $$L=BCE+\;L_{IoU}+\;\alpha \cdot {FP}_{penalty}$$

The model was trained for 50 epochs using the Adam optimiser with a batch size of 8 and a learning rate of 0.0001. For each epoch, the number of training and validation steps was determined by the size of the respective datasets. Data augmentation was applied exclusively to the training set, while the validation set was used in its original, non-augmented form. A summary script for retraining the model is provided in Supplementary Material (Script for retraining the VGG-UNet model.html), and the original model is available in the same Supplementary Material as VGG-UNet.keras.

### 2.2. The Model FabE-Net

The FabE-Net model is also based on a modified U-Net architecture, but it incorporates an EfficientNetV2-S encoder and an enhanced decoder. A pre-trained EfficientNetV2-S model (trained on ImageNet) was used as the backbone encoder, from which feature maps at multiple scales—corresponding to downsampling factors of 2, 4, 8, 16, and 32—were extracted.

Between the encoder and decoder, a series of contextual perception enhancement modules were introduced, including: (1) a Global Context Block, which aggregates global contextual information using attention mechanisms, and (2) an Atrous Spatial Pyramid Pooling (ASPP) module with dilation rates of 1, 6, 12, and 18 to capture features across multiple spatial scales.

The decoder consists of four hierarchically organised spatial resolution recovery blocks, each combining upsampled features with corresponding encoder features via skip connections. Each decoder block is augmented with a Convolutional Block Attention Module (CBAM), which sequentially applies channel attention and spatial attention to adaptively select informative features. In the final stage, a convolutional block followed by bilinear interpolation is applied to restore the output to the original image size. The model architecture diagram is provided in Supplementary Material (Architecture of FabE-Net.png).

To optimise training, a composite loss function was employed, combining the following components:

- Focal Tversky Loss: A modification of the Tversky function designed to emphasise difficult-to-classify pixels, with additional scaling for the positive (foreground) class.
- Balanced Dice Loss: A balanced variant of the Dice loss that accounts for the relative weight of the foreground class.
- Foreground Focal Loss: A modified binary cross-entropy incorporating a focusing factor and weight amplification for positive-class pixels.
- False Positive Penalty: A soft penalty applied to false positive segmentations.

The total loss function was defined as a weighted combination of these components:

(2) $$L=\;0.4\cdot L_{\mathrm{focal tversky}}+0.3\cdot L_{dice}+0.3\cdot L_{\mathrm{foreground focal}}+0.5\cdot L_{\mathrm{false positive penalty}}$$

The model was trained using the AdamW optimiser with an initial learning rate of 0.0001 and a batch size of 8, over 50 epochs. The Dice coefficient, Intersection over Union (IoU), and Recall were used as evaluation metrics to assess the completeness of target object segmentation. A summary script for retraining the model is provided in Supplementary Material (Script for retraining the FabE-Net model.html), and the original model is available in the same Supplementary Material as FabE-Net.pth.

### 2.3. The Model SegFormer

The SegFormer semantic segmentation model is based on a hierarchical transformer architecture and is designed for precise object extraction in images. In this study, the SegFormer-B2 variant was used, pre-trained on the ADE20K dataset and subsequently fine-tuned for the binary segmentation task.

The encoder is a multi-level transformer based on the Mix-Transformer (MiT), which integrates local and global attention mechanisms to enable effective feature extraction across multiple levels of detail. It comprises several stages, each reducing the spatial resolution of feature maps through patching and convolution operations. The early stages of the model focus on processing local image patches, while the deeper stages construct a global contextual representation, enabling the capture of relationships between spatially distant regions. At each successive stage, the number of channels increases, allowing the model to generate progressively more abstract and semantically rich feature representations.

The decoder is a lightweight and computationally efficient module that aggregates multi-level features from the encoder to produce the final segmentation map. A distinctive feature of the SegFormer decoder is the absence of complex upsampling blocks with trainable parameters; instead, it employs simple concatenation and convolution operations, thereby reducing computational complexity and accelerating training.

In the output layer, the model generates logits for each class (in this study, two classes: background and object), which are subsequently converted into probabilities via the softmax function. The model architecture diagram is provided in Supplementary Material (Architecture of SegFormer.png).

Model training was guided by a composite loss function consisting of:

(3) $$L=\;0.4\cdot L_{dice}+0.3\cdot L_{\mathrm{cross entropy}}+0.3\cdot L_{focal\_loss}$$

The model was trained using the SegTrainer class, built upon the Trainer extension from the Hugging Face Transformers library. This custom class redefined the loss function computation to integrate Dice Loss and implemented quality metric calculations. Optimisation was performed using the Adam algorithm with weight decay (AdamW) and an initial learning rate of 0.0001. Training was conducted for 25 epochs with a batch size of 8.

A summary script for model retraining is provided in Supplementary Material (Script for retraining the SegFormer model.html), and the original model is available in the same Supplementary Material within the SegFormer folder. A comparative summary of the architectural features of the models and hyperparameters is presented in Table 1 and Table 2.

## 3. Results

**Figure 4:**
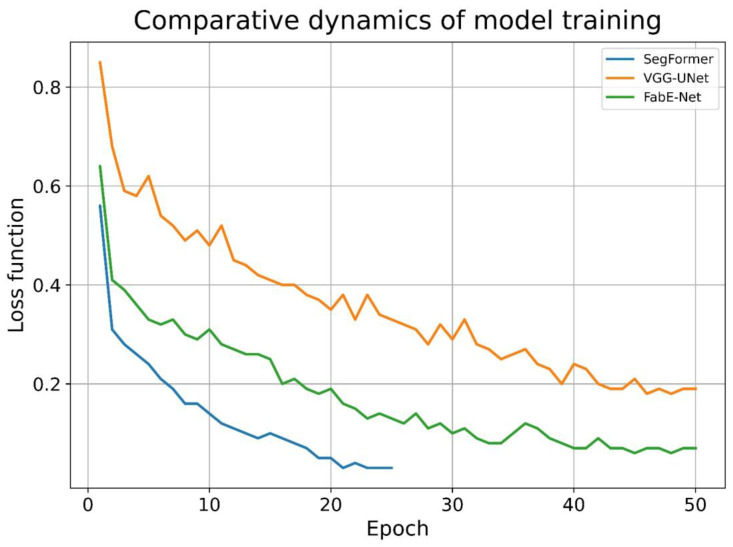
The dynamics of the decrease in the loss function value in the models under study.

During model training, the convergence rate of gradient descent varied across architectures. For the SegFormer model, stable convergence was achieved between the 20th and 30th epochs, whereas the VGG-UNet and FabE-Net models required substantially more iterations, approximately 45 to 60 epochs. Notably, the SegFormer model exhibited the fastest loss function decay, while the VGG-UNet model demonstrated the slowest (Figure 4).

In addition to differences in convergence dynamics, the models also varied substantially in training time. Using identical computing hardware (CPU, 15 GB RAM), the time required for one epoch—comprising approximately 75,000 image pairs—depended on the architecture: for VGG-UNet, training one epoch took an average of ~20 min; for FabE-Net, 35–40 min; and for SegFormer, 90–100 min.

**Figure 5:**
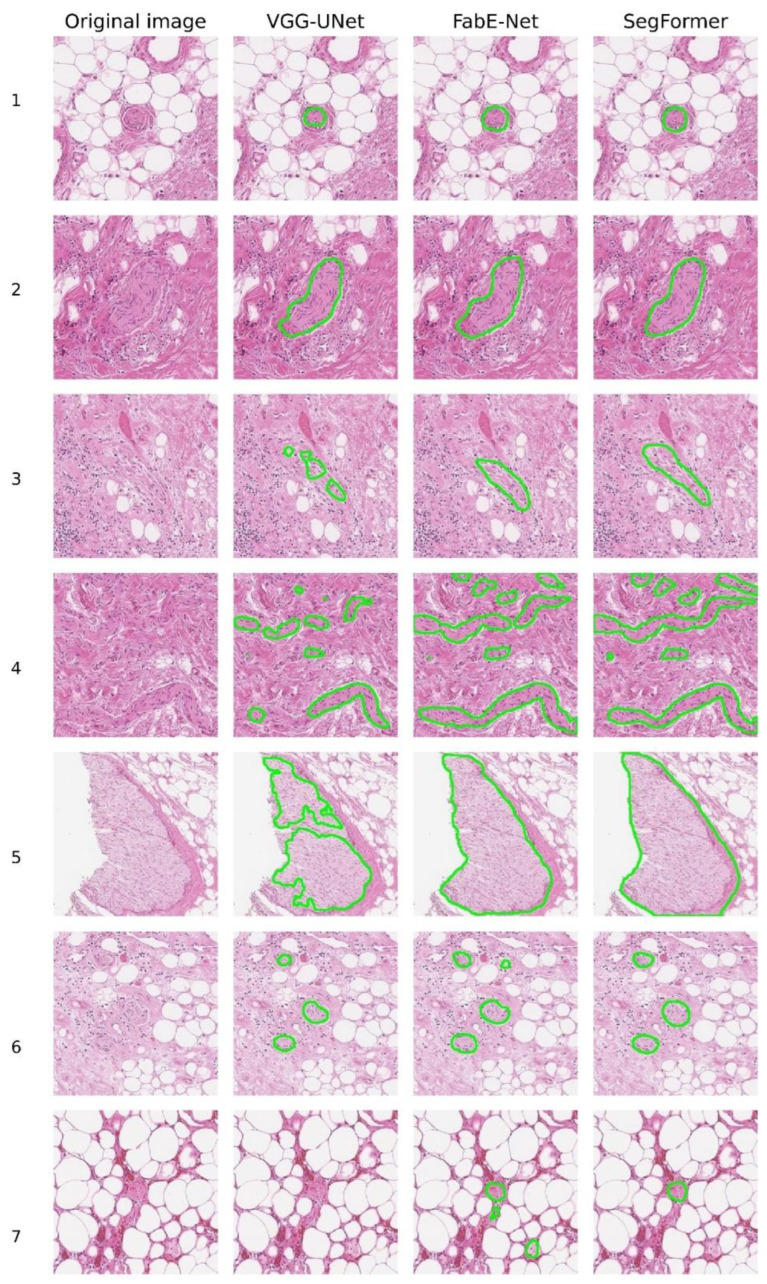
Examples of nerve fibre segmentation by different NNs.

The results of histological image segmentation produced by the different neural network architectures are shown in Figure 5. Qualitative (visual) analysis revealed that the VGG-UNet model often produced incomplete coverage of nerve structures, particularly noticeable in rows 3, 4, and 5. In addition, in some cases (e.g., rows 4 and 7), false-negative segmentation occurred, where nerve fibres were not detected and highlighted by the model.

When comparing the FabE-Net and SegFormer models, it was found that the FabE-Net model is characterised by a more pronounced tendency towards segmentation errors. Among the most frequently observed errors are excessive segmentation, i.e., the selection of a wider area than the actual location of the nerve fibre (e.g., line 2), as well as false positives, where there was no nerve, but the model mistakenly classified other tissues as nerve structures. A typical example is line 7, where FabE-Net recognised macrophages and adipose tissue as nerve tissue.

The image shown in line 4 deserves special attention, as it proved difficult to interpret by all the models under consideration (Figure 5). In this case, all three architectures (VGG-UNet, FabE-Net, and SegFormer) misinterpreted the area of fibrous tissue located in the centre-left region of the image as a nerve fibre. It is also worth noting the time spent on labelling nerve fibres on a complete histological section in svs format with a size of ~600 MB. For the VGG-UNet model, complete marking of the section took about 45 min, for the FabE-Net model—approximately 20 min, while the SegFormer model completed the marking in about 13 min.

To evaluate the effectiveness of the segmentation models, they were retrained on a specialised dataset containing seven myocardial scans, followed by the generation of 5600 labelled images. From this array, 100 images were randomly selected for expert validation so that 60% of the images contained labelled nerve fibres and 40% did not. It is important to note that the selected data was not used during the model training phase.

**Figure 6:**
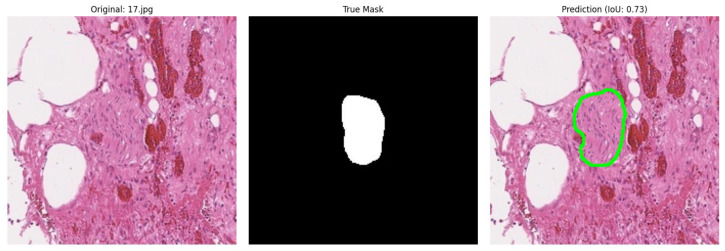
Example of incorrect metric calculation (IoU) during model validation.

During the automatic analysis of the correspondence between expert annotation and model predictions, a significant problem was discovered: in cases where the model correctly identified nerve fibres according to experts, the quantitative metrics (IoU, Dice, etc.) showed low values. This contradiction was explained by the incomplete coincidence of predictions with the original annotation (Figure 6).

To objectively resolve this issue, a group of four independent pathologists who had not participated in the initial marking was brought in. The experts were presented with a series of marked images for evaluation according to the following criteria: Optimal segmentation (complete and accurate nerve isolation); Suboptimal segmentation (partial isolation of the nerve); False positive segmentation; False negative segmentation; True negative segmentation.

The results of the expert validation revealed significant discrepancies in the assessment of segmentation quality. Only in 55% of cases was there complete agreement among all experts, while in 45% of cases their opinions differed. The most frequent source of disagreement (28 out of 45 cases, or 62% of discrepancies) was the subjective boundary between optimal and suboptimal segmentation of nerve fibres.

**Figure 7:**
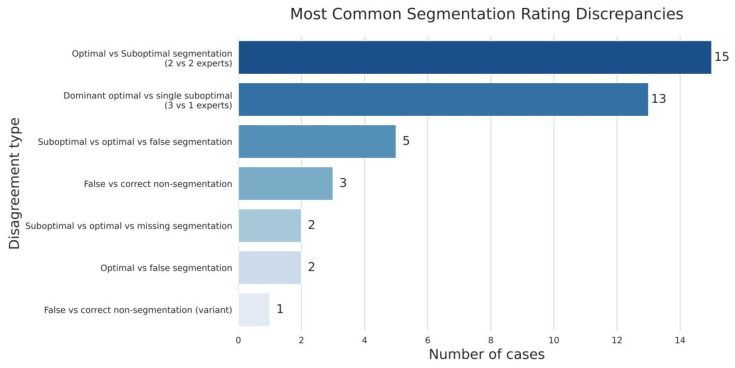
The spectrum of disagreement in expert assessment of nerve fibre segmentation.

Of particular note is the fact that in 17% of cases, the experts demonstrated fundamentally different interpretations–some specialists identified nerve structures in the image, while others denied their presence (Figure 7). This indicates the existence of diagnostically challenging cases where even experienced pathologists cannot reach a consensus on the presence of nerve fibres on histological sections without additional immunohistochemical staining.

To assess inter-expert reliability of the annotations, we calculated agreement metrics across 100 images scored by four independent raters on a 5-point ordinal scale. For each image, individual ratings were tabulated into category frequency distributions, which were then used to compute Fleiss’ κ as a measure of overall categorical agreement and Krippendorff’s α (ordinal) to account for the ordered nature of the scale. Bootstrap resampling (2000 iterations) was employed to estimate 95% confidence intervals. In addition, we quantified the mean observed agreement across all items and the proportion of images with high within-item agreement (P_i_ ≥ 0.80). The analysis demonstrated substantial agreement between experts: Fleiss’ κ was 0.657 [95% CI: 0.573–0.735], Krippendorff’s α (ordinal) was 0.928 [95% CI: 0.897–0.952], the mean observed agreement was 0.768 and 61% of images showed high within-item agreement (P_i_ ≥ 0.80).

To objectively evaluate the performance of various deep learning architectures, a comprehensive analysis of three models was conducted: VGG-UNet, FabE-Net, and SegFormer. The comparison was based on standard segmentation quality metrics, including precision, recall, F1-score, and accuracy, which were calculated as averages based on expert group assessments. The number of true positives was also calculated as the average opinion of the experts.

The study revealed a significant dependence of the evaluation results on the selected segmentation interpretation criteria. When using the most stringent criteria, where only cases of optimal segmentation were considered true positives, the SegFormer model showed the best results, demonstrating satisfactory performance. At the same time, the VGG-UNet and FabE-Net models showed unsatisfactory results under these conditions, indicating their limited effectiveness with this approach to evaluation.

However, the situation changed dramatically when milder evaluation criteria were applied, when both optimal and suboptimal segmentation were considered true positives. In this case, all models showed a significant improvement in metrics, but the degree of this improvement varied considerably. The greatest increase in performance was observed in the VGG-UNet model, which indicates its tendency towards partial rather than complete segmentation of nerve fibres. This conclusion was further confirmed by visual analysis of the model’s results. In contrast, SegFormer showed the smallest increase in metrics when the evaluation criteria were changed, indicating its ability to provide complete segmentation more often. The FabE-Net model occupied an intermediate position, demonstrating good segmentation quality indicators overall, but at the same time, slightly more often than SegFormer, providing partial segmentation of nerve structures (Table 3).

## 4. Discussion

The segmentation of nerves in histological images is an important area of pathomorphological research with a wide range of practical applications, from the routine detection of perineural tumour invasion, which in some cases is considered a significant independent prognostic factor [4], to fundamental studies of organ innervation in various pathological conditions and after denervation interventions [5,6]. This method is also used to reconstruct anatomically accurate three-dimensional models of nerve fibres [7,8], which currently involves significant difficulties associated with the need for laborious manual or semi-automatic segmentation and complex comparison of the location of fibres between different levels of histological sections.

Such technologies are particularly useful in the analysis of large experimental datasets, including studies aimed at developing new approaches to the treatment of pulmonary arterial hypertension (PAH)—a severe progressive disease characterised by remodelling of the pulmonary arteries, increased pressure in the pulmonary circulation, development of chronic pulmonary heart disease and high mortality [9,10]. Since there is currently no complete cure for PAH, and existing methods of treatment either affect only individual links in the pathogenesis or involve heart and lung transplantation [11,12,13], the phenomenon of hyperactivation of the sympathetic nervous system (SNS) in PAH is of particular interest [14,15].

The development of pulmonary artery denervation methods aimed at removing periarterial nerve structures in order to reduce pressure in the pulmonary circulation is a promising direction for treatment [16,17,18]. Endovascular access with preliminary mapping of nerve structures appears to be the most promising [19]. Experimental work in this area includes detailed mapping of pulmonary artery nerve structures in animal models and humans [20,21], but there are no comprehensive studies in humans in normal conditions and with PAH.

A promising approach to solving this problem is the automation of the identification of periarterial nerve structures using artificial intelligence methods, which is particularly relevant when using large datasets obtained both by standard staining methods and by immunohistochemistry.

U-Net-like architectures traditionally occupy a central place in medical imaging, especially when solving problems of histological image segmentation. Their efficiency, resistance to data variability, and wide testing in medical research make them the architectures of choice for such tasks [22,23,24].

With the development of visual transformer architectures (Vision Transformers), their capabilities have been actively studied in medical segmentation tasks. MiT (Mix Transformer) family networks have shown high efficiency in the analysis of histopathological images [25,26]. The self-attention mechanism in transformers allows modelling the global context, identifying dependencies between distant areas of the image and overcoming the local limitations of convolutional networks [27]. This ability to form a holistic view of an image is especially important for histopathology, where spatial relationships between structures and the context of morphological features are critical [28]. However, the use of transformers in this field is still rare, and the results remain controversial. In particular, Yıldız S. et al. showed that combining U-Net-like architectures with Vision Transformer components can yield worse results compared to the classic U-Net [29]. At the same time, a study by Tsiporenko, I. et al. demonstrated that modified transformer architectures, such as SWIN-Supernet, can significantly improve the quality of histological image segmentation compared to U-Net [30].

Our research confirms the hypothesis that attention mechanisms play a key role in achieving high segmentation results. Thus, the FabE-Net architecture, which uses a series of blocks to improve contextual perception, has shown an advantage over the classic U-Net, emphasising the importance of attention layers and multi-level feature processing.

In addition, the SegFormer model also demonstrated superiority over U-Net-like architectures not only in terms of segmentation quality but also in terms of computational efficiency. Thanks to fewer parameters, SegFormer requires fewer resources and reduces the processing time of a complete histological image by 3–3.5 times.

The data obtained allow us to hypothesise that in the tasks of segmenting histopathological images, attention mechanisms play a role not only in integrating the global context but also in compensating for the high morphological variability of tissues. In conditions where the structure and location of cellular elements can vary significantly even within a single pathology, the ability of the model to adaptively redistribute the weight of features depending on their contextual significance becomes a critical factor in segmentation accuracy.

To speed up the segmentation process, we scaled the original images from 1024 × 1024 to 224 × 224 pixels, which significantly reduced the computational load and accelerated processing without losing key morphological features, as confirmed by visual quality control. This approach optimises the performance of models on real data and meets the input requirements of most modern CNN architectures. To our knowledge, such systematic use of reducing the resolution of histological slide tiles in the context of deep learning for segmentation has not yet been described in the literature, which makes the proposed method innovative.

All models considered in the study—including SegFormer, FabE-Net, and VGG-UNet—are available in the Supplementary Material to the article. They can be freely used for retraining and adaptation to new segmentation tasks, as well as partially applied as components for building model ensembles to further improve segmentation quality and reliability of results.

A comparative analysis of three architectures—VGG-UNet, FabE-Net, and SegFormer—showed a clear advantage of the SegFormer model in the task of segmenting nerve structures in histological images. Under strict evaluation criteria, which considered only cases of optimal segmentation as true positives, SegFormer demonstrated the best results across all key metrics (precision, recall, F1-score, accuracy), significantly outperforming VGG-UNet and FabE-Net. Unlike competing architectures, SegFormer performed partial segmentation less frequently and more often provided complete and accurate extraction of target structures, as confirmed by the minimal increase in metrics when switching from strict to more lenient evaluation criteria. This result indicates the model’s high resistance to interpretation variations and its ability to form masks that are more consistent with experts.

The data obtained also revealed a key problem in the segmentation of histological images: the high dependence of the results on the quality of the initial labelling. Expert validation showed that even among experienced pathologists, the level of complete agreement on optimal segmentation is only 55%, with 17% of cases showing fundamental differences in the interpretation of the presence of nerve structures. This emphasises that errors and variability at the annotation stage can significantly affect training samples and, as a result, the final quality of deep learning models. It is important to emphasise that the partial agreement among pathologists reflects not only the inherent subjectivity of histological interpretation but also the methodological limitations of annotation practices. While our dataset relied on expert labelling, the observed discrepancies highlight the potential benefits of consensus-based approaches, where annotations are harmonised across multiple pathologists to minimise individual bias.

It is important to note that even experienced pathologists do not always agree when evaluating the same histological structures. For example, one study noted that the percentage of agreement varied from weak to moderate, especially when analysing micrometastases and small clusters of tumour cells [31]. Significant discrepancies were also observed when counting mitoses in breast tissue—even at the level of individual mitotic figures, where two-thirds agreement among experts was often not achieved [32]. The data indicate that subjective interpretation characteristics, as well as the quality of micro-preparations—including sections, staining, and visualisation—have a particularly critical impact on the accuracy of diagnosing fine structures such as small nerve fibres.

It is also important to note that standard segmentation metrics do not always capture the clinical acceptability of results. For nerve fibres in particular, the precise delineation of boundaries is often limited by preparation artefacts (e.g., tissue tears), endoneurial oedema, or partial loss of the specimen beyond the glass slide. These factors frequently explain cases of partial disagreement among experts, where quantitative metrics produced relatively low scores despite the segmentation being judged as clinically meaningful. Future evaluation frameworks should therefore combine conventional quantitative metrics with consensus-based expert scoring systems to provide a more accurate assessment of clinical relevance.

One promising way to improve the accuracy and consistency of annotation is to use immunohistochemical (IHC) staining to create annotations. The proposed approach can be implemented as follows:

- Two consecutive sections of the same tissue area are digitised—the first with immunohistochemical staining (IHC) for markers specific to the structures of interest (e.g., S100 nerve fibres), the second with the standard haematoxylin-eosin (H&E) method.
- Precise registration (alignment) of the images of the two sections is performed, taking into account microdeformations of the tissue.
- Information about the location of structures identified on the IHC slide is transferred to the corresponding H&E slide.
- The resulting mask is used as a reference label for training segmentation models.

This method minimises subjective errors by annotators and creates the most accurate and reproducible training samples possible. Given the demonstrated superiority of SegFormer over other architectures studied, the integration of these models based on visual transformer architecture with IHC-annotated datasets appears particularly promising. This is likely to lead to the formation of a new standard for the quality of histological image segmentation, increased reproducibility of results, and simplified clinical interpretation, which is particularly important for the development of reliable decision support systems in pathomorphology.

Most previously published studies addressed narrower segmentation tasks, such as identifying myelin, axons, or specific cortical cells, which differ conceptually from our approach focused on nerve fibre segmentation in heterogeneous histological material. Therefore, direct comparison is not entirely appropriate. Our SegFormer model nevertheless demonstrated strong performance (Precision = 0.84, Recall = 0.99, F1 = 0.91, Accuracy = 0.89 for optimal + suboptimal segmentation), clearly surpassing the VGG-UNet and FabE-Net baselines.

By contrast, A. Rasool et al. reported only accuracy values (98–94%), without external expert validation and with signs of annotation inconsistencies [33]. D. Tovbis et al. showed excellent precision, recall, and F1 (~0.91) in fascicle detection, though segmentation was only a secondary step and the dataset was relatively simple [34]. D. Ono et al. achieved high AP and IoU in sural nerve biopsies, but the material was homogeneous and did not involve complex tissue background [35]. Finally, M. M. Fraz demonstrated strong results with FABnet (IoU = 78.4, Dice = 87.9, Accuracy = 96.3), yet their task was restricted to oral cavity tissue, limiting generalizability [36]. Compared to these works, our results highlight the robustness of SegFormer in more challenging and diverse histological settings, supported by evaluation across multiple complementary metrics rather than accuracy alone.

## 5. Conclusions

Attention mechanisms in NN probably play a key role in compensating for the high morphological variability of tissues, ensuring more accurate and consistent segmentation of structures in histological images. In our study, the SegFormer model demonstrated superiority in both nerve fibre segmentation quality and processing speed, significantly outperforming the VGG-UNet and FabE-Net architectures.

The results also highlight the significant impact of annotation variability and subjectivity on the model training process. In this regard, the use of immunohistochemically informed annotation appears to be a promising direction for improving segmentation accuracy and reproducibility.

In addition, we have shown that scaling input images from 1024 × 1024 to 224 × 224 pixels significantly speeds up both the training and mask prediction stages without compromising segmentation quality. This approach can serve as an effective tool for optimising computational resources and is recommended for implementation when developing and training your own histological image segmentation models.

### Study Limitations

A key limitation of this study is the variability in expert annotations. Even among experienced pathologists, complete agreement on the presence or absence of nerve structures was not consistently achieved, and in some cases, fundamental differences in interpretation were observed. This variability inevitably influences the quality of the training data and, consequently, the performance of deep learning models. Future work should consider strategies to mitigate this limitation, including the use of consensus annotations from multiple experts and the integration of immunohistochemistry-based labelling, which may provide more objective reference standards. Although the dataset contained more than 75,000 image–mask pairs, it was ultimately derived from only 64 histological sections obtained from a limited range of tissue types. This restriction may limit the diversity of morphological patterns represented in the training data and raises the possibility of dataset-specific biases. Consequently, the current models should be interpreted with caution when applied to histological images obtained under different staining protocols, tissue origins, or imaging systems. Future work will therefore require external validation on independent datasets encompassing a wider variety of stains, tissue types, and acquisition modalities to confirm the generalizability and robustness of the proposed approach.

## Figures and Tables

**Table 1:** Architectural features of the models.

Model	Backbone	Encoder	Decoder	Attention/Context Modules	Output Layer	Model Metrics Before Training
VGG-UNet	VGG16 (ImageNet pre-trained)	5 convolutional blocks (downsampling to 14 × 14)	4 up-convolutional blocks with skip-connections	BatchNorm + LeakyReLU	Conv2D (1 × 1, sigmoid)	Accuracy: 0.21 Precision: 0.03 Recall: 0.56 IoU: 0.02 Dice: 0.04
FabE-Net	EfficientNet-V2-S (ImageNet pre-trained)	6 encoder stages (32 resolution at bottleneck)	Custom Decoder Blocks with skip-connections	CBAM, Global Context Block, ASPP	Upsample + Conv2D (1 × 1)	Accuracy: 0.27 Precision: 0.03 Recall: 0.68 IoU: 0.03 Dice: 0.06
SegFormer	MiT-B2 (Transformer backbone, ADE20K pre-trained)	Hierarchical transformer encoder (stages: 4, 8, 16, 32 downsampling)	Lightweight MLP decoder	Implicit global attention via transformer	Segmentation head with Conv2D	Accuracy: 0.32 Precision: 0.05 Recall: 0.69 IoU: 0.08 Dice: 0.09

**Table 2:** Training Hyperparameters.

Model	Optimizer	Learning Rate	Batch Size	Epochs
VGG-UNet	Adam	1 × 10^−4^	8	50
FabE-Net	AdamW	1 × 10^−4^	8	50
SegFormer	AdamW (via HF Trainer)	1 × 10^−4^	8	25

**Table 3:** Training metrics of the models under investigation depending on the evaluation strategy.

Metrics		SegFormer	VGG-UNet	FabE-Net
Precision	Only optimal segmentation	0.65	0.19	0.40
	Optimal and suboptimal segmentation	0.84	0.85	0.80
Recall	Only optimal segmentation	0.77	0.20	0.49
	Optimal and suboptimal segmentation	0.99	0.92	0.97
F1 score	Only optimal segmentation	0.88	0.63	0.78
	Optimal and suboptimal segmentation	0.91	0.89	0.88
Accuracy	Only optimal segmentation	0.78	0.49	0.63
	Optimal and suboptimal segmentation	0.89	0.87	0.87

## Data Availability

The original contributions presented in this study are included in the article/Supplementary Material. Further inquiries can be directed to the corresponding author.

## Supplementary Materials

The following supporting information can be downloaded at: https://www.mdpi.com/article/10.3390/diagnostics15182408/s1, Script for preprocessing svs-files to create mask–original image pairs.html.
