# Supplementary material for: A Comparative Analysis of SegFormer, FabE-Net and VGG-UNet Models for the Segmentation of Neural Structures on Histological Sections
Source: Diagnostics (Basel). 2025 Sep 22;15(18):2408. doi: 10.3390/diagnostics15182408 (PMC12468733; doi:10.3390/diagnostics15182408)
Supplement: Supplementary file 1 [file diagnostics-15-02408-s001.zip › Script for retraining the VGG-UNet model.html]

Fine-tuning VGG-UNet Model with Custom Losses


# Fine-tuning a Keras Model with Custom Loss Functions

This script shows how to load a custom VGG-UNet model with custom loss functions and continue training it using a generator.

## 1. Define Custom Loss Functions

```
# Dice loss: measures overlap between prediction and ground truth
def dice_loss(y_true, y_pred, smooth=1e-6):
    y_true_f = K.flatten(y_true)
    y_pred_f = K.flatten(y_pred)
    intersection = K.sum(y_true_f * y_pred_f)
    return 1 - (2. * intersection + smooth) / (K.sum(y_true_f) + K.sum(y_pred_f) + smooth)

# Binary crossentropy + dice loss
def combined_loss(y_true, y_pred):
    return BinaryCrossentropy()(y_true, y_pred) + dice_loss(y_true, y_pred)

# Intersection over Union (IoU) loss
def iou_loss(y_true, y_pred, smooth=1e-6):
    y_true_f = K.flatten(y_true)
    y_pred_f = K.flatten(y_pred)
    intersection = K.sum(y_true_f * y_pred_f)
    union = K.sum(y_true_f) + K.sum(y_pred_f) - intersection
    iou = (intersection + smooth) / (union + smooth)
    return 1 - iou

# Penalize false positives to reduce over-segmentation
def false_positive_penalty(y_true, y_pred, alpha=2.0):
    y_true_f = K.flatten(y_true)
    y_pred_f = K.flatten(y_pred)
    false_positives = y_pred_f * (1 - y_true_f)
    penalty = K.sum(false_positives) / (K.sum(K.ones_like(y_true_f)) + K.epsilon())
    return alpha * penalty

# Enhanced IoU loss combining BCE, IoU, and false positive penalty
def enhanced_iou_loss(y_true, y_pred):
    bce = BinaryCrossentropy()(y_true, y_pred)
    iou = iou_loss(y_true, y_pred)
    fp_penalty = false_positive_penalty(y_true, y_pred, alpha=2.5)
    return bce + iou + fp_penalty
```

## 2. Load the Pretrained Model with Custom Losses

```
custom_objects = {
    'dice_loss': dice_loss,
    'combined_loss': combined_loss,
    'iou_loss': iou_loss,
    'false_positive_penalty': false_positive_penalty,
    'enhanced_iou_loss': enhanced_iou_loss
}

model = load_model(path_to_model, custom_objects=custom_objects)
```

## 3. Data Generator with Optional Augmentation

```
def data_generator(img_paths, mask_paths, batch_size=32, target_size=(224, 224), augment=False):
    num_samples = len(img_paths)
    indices = np.arange(num_samples)

    while True:
        np.random.shuffle(indices)
        for i in range(0, num_samples, batch_size):
            batch_indices = indices[i:i + batch_size]
            batch_images = []
            batch_masks = []

            for idx in batch_indices:
                img = cv2.imread(img_paths[idx])
                img = cv2.cvtColor(img, cv2.COLOR_BGR2RGB)
                img = cv2.resize(img, target_size)
                img = preprocess_input(img)

                mask = cv2.imread(mask_paths[idx], cv2.IMREAD_GRAYSCALE)
                mask = cv2.resize(mask, target_size)
                mask = (mask > 127).astype(np.float32)
                mask = np.expand_dims(mask, axis=-1)

                if augment:
                    if np.random.rand() < 0.5:
                        img = np.fliplr(img)
                        mask = np.fliplr(mask)
                    if np.random.rand() < 0.5:
                        img = np.flipud(img)
                        mask = np.flipud(mask)
                    if np.random.rand() < 0.3:
                        angle = np.random.uniform(-15, 15)
                        M = cv2.getRotationMatrix2D((target_size[0] / 2, target_size[1] / 2), angle, 1)
                        img = cv2.warpAffine(img, M, target_size, borderMode=cv2.BORDER_REFLECT)
                        mask = cv2.warpAffine(mask, M, target_size, borderMode=cv2.BORDER_REFLECT)
                        mask = np.expand_dims(mask, axis=-1)

                if img.shape != (target_size[0], target_size[1], 3):
                    print(f"Incorrect image size: {img.shape} -> {img_paths[idx]}")
                    continue
                if mask.shape != (target_size[0], target_size[1], 1):
                    print(f"Incorrect mask size: {mask.shape} -> {mask_paths[idx]}")
                    continue

                batch_images.append(img)
                batch_masks.append(mask)

            if len(batch_images) == 0:
                continue

            yield np.array(batch_images, dtype=np.float32), np.array(batch_masks, dtype=np.float32)
```

## 4. Train/Validation Generators Setup

```
train_gen = data_generator(train_img_paths, train_mask_paths, batch_size=8, augment=True)
val_gen = data_generator(val_img_paths, val_mask_paths, batch_size=8)
validation_steps = len(val_img_paths) // 8
train_steps = len(train_img_paths) // 8
```

## 5. Fine-tuning Function

```
def fine_tune_model(model, train_gen, val_gen, train_steps, val_steps, epochs=10, learning_rate=1e-4):
    """
    Fine-tunes an existing model with new data.
    Args:
        model: Loaded Keras model.
        train_gen: Generator for training data.
        val_gen: Generator for validation data.
        train_steps: Number of training steps per epoch.
        val_steps: Number of validation steps per epoch.
        epochs: Number of fine-tuning epochs.
        learning_rate: Learning rate for optimizer.
    """
    model.compile(optimizer=tf.keras.optimizers.Adam(learning_rate=learning_rate),
                  loss=enhanced_iou_loss,
                  metrics=['accuracy'])

    history = model.fit(
        train_gen,
        steps_per_epoch=train_steps,
        validation_data=val_gen,
        validation_steps=val_steps,
        epochs=epochs
    )

    return history
```

## 6. Example Fine-tuning Call

```
fine_tune_model(model, train_gen, val_gen, train_steps, validation_steps, epochs=5, learning_rate=1e-5)
```
