# Supplementary material for: A Comparative Analysis of SegFormer, FabE-Net and VGG-UNet Models for the Segmentation of Neural Structures on Histological Sections
Source: Diagnostics (Basel). 2025 Sep 22;15(18):2408. doi: 10.3390/diagnostics15182408 (PMC12468733; doi:10.3390/diagnostics15182408)
Supplement: Supplementary file 1 [file diagnostics-15-02408-s001.zip › Script for retraining the FabE-Net model.html]

FabE-Net Fine-Tuning Guide


# Fine-Tuning FabE-Net (FABNetAttEfficientNet)

This page demonstrates a method for tuning the **FabE-Net** model on additional data for the nerve fiber segmentation task.

## 1. Environment Setup

Ensure the following libraries are installed: PyTorch, Albumentations, OpenCV, tqdm.

```
pip install torch torchvision albumentations opencv-python tqdm
```

## 2. Dataset Preparation

Organize your dataset with matching filenames in the following structure:

- `/path/to/dataset/images` — input images (.jpg/.png)
- `/path/to/dataset/masks` — corresponding binary masks

Images and masks must have identical filenames.

## 3. Loss Function

We use a composite custom loss function `EnhancedSegmentationLossFP` combining Focal Tversky, Balanced Dice, Foreground Focal, and False Positive Penalty.

```
import torch
import torch.nn as nn
import torch.nn.functional as F

class EnhancedSegmentationLossFP(nn.Module):
    def __init__(self, smooth=1e-6, alpha=0.25, gamma=2.0, beta=4.5):
        super().__init__()
        self.smooth = smooth
        self.alpha = alpha
        self.gamma = gamma
        self.beta = beta
        self.bce = nn.BCEWithLogitsLoss(reduction='none')

    def focal_tversky_loss(self, pred, target):
        pred = torch.sigmoid(pred)
        pos_weight = torch.clamp(1.0 / (target.sum(dim=(2,3), keepdim=True)) * self.beta, 1.0, 10.0)
        tp = (pred * target * pos_weight).sum()
        fp = (pred * (1 - target)).sum()
        fn = ((1 - pred) * target * pos_weight).sum()
        tversky = (tp + self.smooth) / (tp + self.alpha * fp + (1 - self.alpha) * fn + self.smooth)
        return torch.pow((1 - tversky), 1 / self.gamma)

    def balanced_dice_loss(self, pred, target):
        pred = torch.sigmoid(pred)
        pos_weight = torch.clamp(1.0 / (target.sum(dim=(2,3), keepdim=True)) * self.beta, 1.0, 10.0)
        intersection = (pred * target * pos_weight).sum()
        union = (pred * pos_weight).sum() + target.sum()
        return 1 - (2. * intersection + self.smooth) / (union + self.smooth)

    def foreground_focal_loss(self, pred, target):
        bce = self.bce(pred, target)
        pt = torch.exp(-bce)
        focal_weight = (1 - pt) ** self.gamma
        foreground_weight = target * (self.beta - 1) + 1
        return (focal_weight * bce * foreground_weight).mean()

    def soft_negative_penalty(self, pred, target):
        pred = torch.sigmoid(pred)
        fp = (pred * (1 - target)).mean()
        return fp

    def forward(self, outputs, target):
        pred = outputs['main']
        ft = self.focal_tversky_loss(pred, target)
        dice = self.balanced_dice_loss(pred, target)
        ff = self.foreground_focal_loss(pred, target)
        fp_penalty = 0.5 * self.soft_negative_penalty(pred, target)
        loss = 0.4 * ft + 0.3 * dice + 0.3 * ff + fp_penalty
        return loss, {
            'loss': loss.item(),
            'focal_tversky': ft.item(),
            'balanced_dice': dice.item(),
            'foreground_focal': ff.item(),
            'false_positive_penalty': fp_penalty.item()
        }
```

## 4. Model Loading

Load a pretrained model or instantiate from scratch:

```
model = torch.load(path_to_model, map_location=device)
```

## 5. Data Augmentation and Loader

Train/validation datasets are split with augmentation applied only to the training set.

```
train_loader, val_loader = prepare_dataloaders(
    img_dir='/path/to/images',
    mask_dir='/path/to/masks',
    batch_size=8
)
```

## 6. Training Loop

This function handles training and validation per epoch. It tracks loss, Dice, IoU, and Recall.

```
for epoch in range(1, n_epoch + 1):
    print(f"\nEpoch {epoch}")
    train_metrics = train_one_epoch(model, train_loader, optimizer, criterion, device)
    val_metrics = validate(model, val_loader, criterion, device)

    print(f"Train: Loss={train_metrics['loss']:.4f}, Dice={train_metrics['dice']:.4f}, IoU={train_metrics['iou']:.4f}, Recall={train_metrics['recall']:.4f}")
    print(f"Valid: Loss={val_metrics['loss']:.4f}, Dice={val_metrics['dice']:.4f}, IoU={val_metrics['iou']:.4f}, Recall={val_metrics['recall']:.4f}")
```

## 7. Optimizer and Scheduler

Use `AdamW` optimizer and optionally a learning rate scheduler.

```
optimizer = torch.optim.AdamW(model.parameters(), lr=1e-4)
# scheduler = torch.optim.lr_scheduler.StepLR(optimizer, step_size=5, gamma=0.5)
```

## 8. Saving the Model

After training:

```
torch.save(model.state_dict(), 'FabE-Net_finetuned.pth')
```

## 9. Evaluation

Apply the model to new images using `torch.sigmoid` and post-process as needed:

```
model.eval()
with torch.no_grad():
    image = ...  # preprocessed input tensor
    pred = torch.sigmoid(model(image)['main'])
    binary_mask = (pred > 0.5).float()
```
