# Supplementary material for: A Comparative Analysis of SegFormer, FabE-Net and VGG-UNet Models for the Segmentation of Neural Structures on Histological Sections
Source: Diagnostics (Basel). 2025 Sep 22;15(18):2408. doi: 10.3390/diagnostics15182408 (PMC12468733; doi:10.3390/diagnostics15182408)
Supplement: Supplementary file 1 [file diagnostics-15-02408-s001.zip › Script for retraining the SegFormer model.html]

Segformer Fine-Tuning Guide


# Fine-Tuning the Segformer Model for Semantic Segmentation

## 1. Model Initialization

This section shows how to load a pre-trained Segformer model with an adjusted number of output classes for segmenting nerve fibers in histological images.

```
model = SegformerForSemanticSegmentation.from_pretrained(
    model_path,
    num_labels=2,
    ignore_mismatched_sizes=True
)
```

## 2. Custom Trainer Class

We define a subclass of `Trainer` to customize the loss calculation and evaluation metrics:

```
class SegTrainer(Trainer):
    def compute_loss(self, model, inputs, return_outputs=False, **kwargs):
        labels = inputs.pop("labels")
        outputs = model(**inputs)
        logits = outputs.logits

        # Resize labels to match logits size (nearest interpolation)
        labels = F.interpolate(labels.unsqueeze(1).float(), size=logits.shape[2:], mode="nearest").squeeze(1).long()

        loss = dice_loss(logits, labels)
        return (loss, outputs) if return_outputs else loss

    def compute_metrics(self, eval_pred):
        logits, labels = eval_pred
        preds = torch.tensor(logits).softmax(dim=1).argmax(dim=1).numpy()
        labels = labels.astype(np.int32)

        iou_scores = []
        for p, t in zip(preds, labels):
            iou_scores.append(jaccard_score(t.flatten(), p.flatten(), average='binary'))
        mean_iou = np.mean(iou_scores)
        return {"mean_iou": mean_iou}
```

## 3. Dataset Class

Dataset class handles loading images and masks, with optional augmentations.

```
class HistologySegDataset(Dataset):
    def __init__(self, image_dir, mask_dir, image_list, processor, augment=False):
        self.image_dir = image_dir
        self.mask_dir = mask_dir
        self.image_list = image_list
        self.processor = processor
        self.augment = augment

        self.augmentations = transforms.Compose([
            transforms.RandomHorizontalFlip(),
            transforms.RandomVerticalFlip(),
            transforms.RandomRotation(20),
            transforms.ColorJitter(brightness=0.2, contrast=0.2, saturation=0.2),
            transforms.Resize((224, 224))
        ])
        self.no_aug = transforms.Resize((224, 224))

    def __len__(self):
        return len(self.image_list)

    def __getitem__(self, idx):
        image_path = os.path.join(self.image_dir, self.image_list[idx])
        mask_path = os.path.join(self.mask_dir, self.image_list[idx])

        image = Image.open(image_path).convert("RGB")
        mask = Image.open(mask_path).convert("L")

        if self.augment:
            seed = np.random.randint(2147483647)
            torch.manual_seed(seed)
            image = self.augmentations(image)
            torch.manual_seed(seed)
            mask = self.augmentations(mask)
        else:
            image = self.no_aug(image)
            mask = self.no_aug(mask)

        mask = np.array(mask)
        mask = (mask > 127).astype(np.uint8)

        inputs = self.processor(image, return_tensors="pt")
        inputs["labels"] = torch.tensor(mask, dtype=torch.long).unsqueeze(0)

        inputs["pixel_values"] = inputs["pixel_values"].squeeze(0)
        inputs["labels"] = inputs["labels"].squeeze(0)
        return inputs
```

## 4. Loss Functions

We use a combination of Dice loss, Focal loss, and Cross-Entropy loss for better training stability and segmentation accuracy.

```
def dice_loss(preds, targets, smooth=1):
    preds = F.softmax(preds, dim=1)[:, 1]  # class 1 (foreground)
    targets = targets.float()
    intersection = (preds * targets).sum()
    union = preds.sum() + targets.sum()
    dice = (2. * intersection + smooth) / (union + smooth)
    return 1 - dice

def focal_loss(logits, targets, gamma=2.0, alpha=0.25):
    ce_loss = F.cross_entropy(logits, targets, reduction='none')
    pt = torch.exp(-ce_loss)
    focal = alpha * (1 - pt) ** gamma * ce_loss
    return focal.mean()

def combined_loss(logits, targets, alpha=0.25, gamma=2.0, weights=(0.4, 0.3, 0.3)):
    bce = F.cross_entropy(logits, targets)
    dice = dice_loss(logits, targets)
    focal = focal_loss(logits, targets, gamma=gamma, alpha=alpha)
    return weights[0] * dice + weights[1] * bce + weights[2] * focal
```

## 5. Metric Computation

IoU metric is calculated during evaluation to monitor segmentation performance on the validation set.

```
def compute_iou(preds, labels, threshold=0.5, smooth=1e-6):
    probs = torch.softmax(preds, dim=1)[:, 1]
    preds_bin = (probs > threshold).float()
    labels = labels.float()

    intersection = (preds_bin * labels).sum(dim=(1, 2))
    union = preds_bin.sum(dim=(1, 2)) + labels.sum(dim=(1, 2)) - intersection

    iou = (intersection + smooth) / (union + smooth)
    return iou.mean().item()

def compute_metrics(eval_pred):
    logits, labels = eval_pred
    iou = compute_iou(torch.tensor(logits), torch.tensor(labels))
    return {"mean_iou": iou}
```

## 6. Training Arguments and Training Process

We configure the training parameters and start the fine-tuning process with the custom trainer.

```
training_args = TrainingArguments(
    output_dir="./segformer-histo",
    per_device_train_batch_size=8,
    per_device_eval_batch_size=4,
    num_train_epochs=25,
    logging_steps=50,
    logging_dir="./logs",
    report_to="none",
    run_name="segformer-histo-run"
)

trainer = SegTrainer(
    model=model,
    args=training_args,
    train_dataset=train_dataset,
    eval_dataset=val_dataset,
    compute_metrics=compute_metrics,
)

trainer.train()
```
