# Supplementary material for: A Comparative Analysis of SegFormer, FabE-Net and VGG-UNet Models for the Segmentation of Neural Structures on Histological Sections
Source: Diagnostics (Basel). 2025 Sep 22;15(18):2408. doi: 10.3390/diagnostics15182408 (PMC12468733; doi:10.3390/diagnostics15182408)
Supplement: Supplementary file 1 [file diagnostics-15-02408-s001.zip › Script for preprocessing svs-files to create mask-original image pairs.html]

Code Visualization


# Histology Image Normalization

```
def rgb_to_od(img):
    """Convert RGB to Optical Density (OD) with zero protection"""
    img = np.clip(img, 1, 255).astype(np.float32)
    return -np.log(img / 255)

def od_to_rgb(od):
    """Convert OD back to RGB"""
    od = np.clip(od, a_min=1e-6, a_max=None)
    return (255 * np.exp(-od)).astype(np.uint8)

def get_stain_matrix(img, beta=0.15, alpha=1):
    """Calculate stain matrix (2x3) via SVD"""
    od = rgb_to_od(img).reshape((-1, 3))
    od = od[np.any(od > beta, axis=1)]

    _, _, V = np.linalg.svd(od, full_matrices=False)
    V = V[:2, :]

    if V[0, 0] < 0: V[0] *= -1
    if V[1, 0] < 0: V[1] *= -1

    return V

def normalize_histology_image(tile, target_image):
    """Normalize histology images by Macenko method"""
    tile = exposure.rescale_intensity(tile, out_range=(0, 255)).astype(np.uint8)
    target = exposure.rescale_intensity(target_image, out_range=(0, 255)).astype(np.uint8)

    W_target = get_stain_matrix(target)
    W_source = get_stain_matrix(tile)

    od = rgb_to_od(tile).reshape((-1, 3))
    C = od @ np.linalg.pinv(W_source)

    max_C_source = np.percentile(C, 99, axis=0)
    max_C_target = np.percentile(
        rgb_to_od(target).reshape((-1, 3)) @ np.linalg.pinv(W_target),
        99,
        axis=0
    )
    C = C * (max_C_target / max_C_source)

    od_norm = C @ W_target
    return od_to_rgb(od_norm.reshape(tile.shape))

def normalize_folder(input_folder, target_image_path, output_folder):
    os.makedirs(output_folder, exist_ok=True)

    target = cv2.imread(target_image_path)
    target = cv2.cvtColor(target, cv2.COLOR_BGR2RGB)

    for fname in tqdm(os.listdir(input_folder)):
        if not fname.lower().endswith(('.png', '.jpg', '.jpeg', '.tif')):
            continue

        img_path = os.path.join(input_folder, fname)
        img = cv2.imread(img_path)
        img = cv2.cvtColor(img, cv2.COLOR_BGR2RGB)

        try:
            normalized = normalize_histology_image(img, target)
            save_path = os.path.join(output_folder, fname)
            cv2.imwrite(save_path, normalized)
        except Exception as e:
            print(f"Error processing {fname}: {e}")
```

# XML Parsing for SVS Slide Annotation

```
from lxml import etree
import numpy as np

def parse_xml_with_layers(xml_content):
    """Parse XML with grouping by layers and flat list of all regions."""
    if isinstance(xml_content, BeautifulSoup):
        xml_content = str(xml_content)

    if isinstance(xml_content, str):
        xml_content = xml_content.encode('utf-8')

    parser = etree.XMLParser(recover=True, remove_blank_text=True)
    try:
        tree = etree.fromstring(xml_content, parser=parser)
    except etree.XMLSyntaxError as e:
        print(f"XML parsing error: {e}")
        return {'Layers': {}, 'all_regions': {}}

    Layers = {}
    all_regions = {}

    for annotation in tree.xpath('//Annotation'):
        layer_id = annotation.get('Id')
        layer_name = annotation.get('Name', f"Layer_{layer_id}")
        layer_regions = {}

        for region in annotation.xpath('.//Region'):
            try:
                region_id = region.get('Id')
                if not region_id:
                    continue

                vertices = []
                for vertex in region.xpath('.//Vertex'):
                    try:
                        x = float(vertex.get('X', '0'))
                        y = float(vertex.get('Y', '0'))
                        vertices.append([x, y])
                    except (TypeError, ValueError):
                        continue

                if not vertices:
                    continue

                region_data = {
                    'AreaMicrons': float(region.get('AreaMicrons', '0')),
                    'Vertices': np.array(vertices)
                }

                layer_regions[region_id] = region_data
                all_regions[region_id] = region_data

            except Exception as e:
                print(f"Error processing region {region.get('Id')}: {e}")
                continue

        Layers[layer_name] = layer_regions
    return {'Layers': Layers, 'all_regions': all_regions}
```

# Annotated Image Pair Plotting

```
def plot_annotated_images_enhanced(slide_path, dic, number, layer, margin=100, size_of_read=512,
                                 monomorphic_size=None,
                                 linear_augmentation=None,
                                 save_to_folders=None):
    """Enhanced function with saving and batch processing"""

    if isinstance(number, (list, tuple)):
        for num in tqdm(number, desc="Processing numbers"):
            try:
                _process_single_number(slide_path, dic, num, layer, margin, size_of_read,
                                       monomorphic_size, linear_augmentation, save_to_folders)
            except Exception as e:
                print(f"Error processing number {num}: {str(e)}")
        return

    _process_single_number(slide_path, dic, number, layer, margin, size_of_read,
                          monomorphic_size, linear_augmentation, save_to_folders)

def _process_single_number(slide_path, dic, number, layer, margin, size_of_read,
                         monomorphic_size, linear_augmentation, save_to_folders):
    slide = openslide.OpenSlide(slide_path)
    try:
        if save_to_folders is not None:
            original_path, mask_path = save_to_folders
            os.makedirs(original_path, exist_ok=True)
            os.makedirs(mask_path, exist_ok=True)

        main_data = list(dic.values())[layer].get(str(number), {})
        if not main_data or 'Vertices' not in main_data:
            print(f"Error: number {number} in layer {layer} missing 'Vertices'")
            return

        main_vertices = main_data['Vertices']

        min_x = int(min(main_vertices[:, 0])) - margin
        max_x = int(max(main_vertices[:, 0])) + margin
        min_y = int(min(main_vertices[:, 1])) - margin
        max_y = int(max(main_vertices[:, 1])) + margin

        original_width = max_x - min_x
        original_height = max_y - min_y

        squ = max(original_width, original_height)
        if squ < size_of_read:
            squ = size_of_read

        base_min_x = min_x - (squ - original_width) // 2
        base_min_y = min_y - (squ - original_height) // 2

        n_images = 1
        offsets = [(0, 0)]

        if linear_augmentation is not None:
            n_images, min_offset, max_offset = linear_augmentation
            for _ in range(n_images - 1):
                offset_x = random.randint(min_offset, max_offset)
                offset_y = random.randint(min_offset, max_offset)
                offsets.append((offset_x, offset_y))

        if monomorphic_size is not None:
            target_w, target_h = monomorphic_size
            scale_x = target_w / squ
            scale_y = target_h / squ
        else:
            target_w, target_h = squ, squ
            scale_x, scale_y = 1, 1

        if save_to_folders is None:
            fig_cols = 3
            fig_rows = n_images
            fig, axes = plt.subplots(fig_rows, fig_cols,
                                   figsize=(6*fig_cols, 6*fig_rows))

            if n_images == 1:
                axes = axes.reshape(1, -1)

        for img_idx, (offset_x, offset_y) in enumerate(offsets):
            new_min_x = base_min_x + offset_x
            new_min_y = base_min_y + offset_y

            img = slide.read_region((new_min_x, new_min_y), 0, (squ, squ)).convert("RGB")

            if monomorphic_size is not None:
                img = img.resize((target_w, target_h), Image.LANCZOS)

            img_with_lines = img.copy()
            draw = ImageDraw.Draw(img_with_lines)

            mask = Image.new("L", (target_w, target_h), 0)
            draw_mask = ImageDraw.Draw(mask)

            regions_found = 0

            for current_layer in dic.values():
                for current_number, current_data in current_layer.items():
                    if 'Vertices' not in current_data:
                        continue

                    current_vertices = current_data['Vertices']
                    points = [(int((px - new_min_x) * scale_x),
                              int((py - new_min_y) * scale_y))
                             for px, py in zip(current_vertices[:, 0], current_vertices[:, 1])]

                    if any(0 <= px < target_w and 0 <= py < target_h for px, py in points):
                        regions_found += 1
                        draw.line(points + [points[0]], fill="red", width=max(1, int(2*min(scale_x, scale_y))))
                        draw_mask.polygon(points, fill=255)

            base_filename = f"{number}_layer{layer}_img{img_idx}"
            if offset_x != 0 or offset_y != 0:
                base_filename += f"_x{offset_x}y{offset_y}"

            if save_to_folders is not None:
                original_path, mask_path = save_to_folders

                img.save(os.path.join(original_path, f"{slide_path.split('.svs')[0][-7:]}_{base_filename}.jpg".replace('/', '_')), quality=95)
                mask.save(os.path.join(mask_path, f"{slide_path.split('.svs')[0][-7:]}_{base_filename}.jpg".replace('/', '_')), quality=95)
            else:
                axes[img_idx, 0].imshow(img)
                axes[img_idx, 0].set_title(f'Original {img_idx+1}' + (f'\nOffset: ({offset_x},{offset_y})' if img_idx > 0 else ''))
                axes[img_idx, 0].axis('off')

                axes[img_idx, 1].imshow(img_with_lines)
                axes[img_idx, 1].set_title(f'Annotated {img_idx+1}\n({regions_found} regions)')
                axes[img_idx, 1].axis('off')

                axes[img_idx, 2].imshow(mask, cmap='gray')
                axes[img_idx, 2].set_title(f'Mask {img_idx+1}')
                axes[img_idx, 2].axis('off')

        if save_to_folders is None:
            plt.tight_layout()
            plt.show()

    except Exception as e:
        print(f"Critical error processing number {number}: {str(e)}")
```
