# Supplementary material for: A Comparative Analysis of SegFormer, FabE-Net and VGG-UNet Models for the Segmentation of Neural Structures on Histological Sections
Source: Diagnostics (Basel). 2025 Sep 22;15(18):2408. doi: 10.3390/diagnostics15182408 (PMC12468733; doi:10.3390/diagnostics15182408)
Supplement: Supplementary file 1 [file diagnostics-15-02408-s001.zip › Formulas.html]

Loss Functions


Loss Functions

| Name | Formula | Description |
| Binary Cross-Entropy (BCE) | \[ L\_{\text{BCE}} = - \frac{1}{N} \sum\_{i=1}^{N} \Big[ y\_i \log(p\_i) + (1-y\_i)\log(1-p\_i) \Big] \] | Standard loss for binary classification; penalizes incorrect predictions equally. |
| Intersection over Union (IoU Loss) | \[ L\_{\text{IoU}} = 1 - \frac{\sum\_{i=1}^{N} y\_i p\_i}{\sum\_{i=1}^{N} y\_i + p\_i - y\_i p\_i} \] | Optimizes overlap between predicted and true regions; robust to class imbalance. |
| False Positive Penalty | \[ L\_{\text{FP}} = \frac{\sum\_{i=1}^{N} (1-y\_i) p\_i}{\sum\_{i=1}^{N} y\_i + \epsilon} \] | Explicitly penalizes false positives; useful for tasks requiring high precision. |
| Focal Tversky Loss | \[ TI = \frac{\sum\_{i=1}^{N} y\_i p\_i}{\sum\_{i=1}^{N} y\_i p\_i + \alpha \sum\_{i=1}^{N} y\_i (1-p\_i) + \beta \sum\_{i=1}^{N} (1-y\_i)p\_i} \] \[ L\_{\text{FTL}} = (1 - TI)^{\gamma} \] | Extension of Tversky loss with focus on hard-to-classify pixels; balances false negatives and false positives. |
| Balanced Dice Loss | \[ L\_{\text{Dice}} = 1 - \frac{2 \sum\_{i=1}^{N} y\_i p\_i}{\sum\_{i=1}^{N} y\_i + \sum\_{i=1}^{N} p\_i} \] \[ L\_{\text{Balanced Dice}} = 1 - \frac{(1+\lambda)\sum\_{i=1}^{N} y\_i p\_i}{\lambda \sum\_{i=1}^{N} y\_i + \sum\_{i=1}^{N} p\_i} \] | Dice loss variant that adds a weight to foreground class; improves segmentation in imbalanced datasets. |
| Foreground Focal Loss | \[ L\_{\text{FFL}} = - \frac{1}{N} \sum\_{i=1}^{N} \alpha y\_i (1-p\_i)^{\gamma} \log(p\_i) - (1-\alpha)(1-y\_i) p\_i^{\gamma} \log(1-p\_i) \] | Modified BCE with focusing factor and higher weight on foreground pixels. |

### Notation

\( y\_i \in \{0,1\} \) — ground-truth label for pixel \( i \).

\( p\_i \in [0,1] \) — predicted probability of pixel \( i \) belonging to the foreground class.

\( N \) — number of pixels.

\( \alpha, \beta, \gamma \) — hyperparameters (weighting coefficients, focusing factors).

\( \lambda \) — balancing coefficient for Dice loss.

\( \epsilon \) — small constant for numerical stability.
