# Supplementary figures and images for: A Comparative Analysis of SegFormer, FabE-Net and VGG-UNet Models for the Segmentation of Neural Structures on Histological Sections
Source: Diagnostics (Basel). 2025 Sep 22;15(18):2408. doi: 10.3390/diagnostics15182408 (PMC12468733; doi:10.3390/diagnostics15182408)

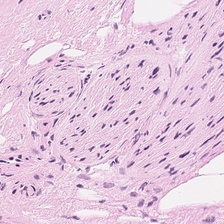

Supplement: Supplementary file 1 [file diagnostics-15-02408-s001.zip › target_image.jpg]
